# Liquid Biopsy as a New Tool for Diagnosis and Monitoring in Renal Cell Carcinoma

**DOI:** 10.3390/cancers17091442

**Published:** 2025-04-25

**Authors:** Giuseppe Stefano Netti, Federica De Luca, Valentina Camporeale, Javeria Khalid, Giorgia Leccese, Dario Troise, Francesca Sanguedolce, Giovanni Stallone, Elena Ranieri

**Affiliations:** 1 Unit of Clinical Pathology, Department of Medical and Surgical Sciences, University of Foggia–University Hospital “Policlinico Riuniti”, Viale Luigi Pinto, 71122 Foggia, Italy; 2 Center for Research and Innovation in Medicine (CREATE), Department of Medical and Surgical Sciences, University of Foggia–University Hospital “Policlinico Riuniti”, Viale Luigi Pinto, 71122 Foggia, Italy; 3 Unit of Nephrology, Dialysis and Transplantation, Advanced Research Center on Kidney Aging (A.R.K.A.), Department of Medical and Surgical Sciences, University of Foggia–University Hospital “Policlinico Riuniti”, Viale Luigi Pinto, 71122 Foggia, Italy; 4 Renal Medicine and Baxter Novum, Department of Clinical Science, Intervention and Technology, Karolinska Institutet, 141 52 Stockholm, Sweden; 5 Unit of Pathology, Department of Clinical and Experimental Medicine, University of Foggia–University Hospital “Policlinico Riuniti”, Viale Luigi Pinto, 71122 Foggia, Italy

**Keywords:** renal cell carcinoma, liquid biopsy, microRNA, exosomes, extracellular vesicles, circulating tumor cells, circulating tumor DNA

## Abstract

Renal cell carcinoma (RCC) presents a significant diagnostic challenge, particularly in small renal masses. The search for non-invasive screening methods and biomarkers has directed research toward liquid biopsy, which focuses on microRNAs (miRNAs), exosomes, and circulating tumor cells (CTCs). miRNAs are small non-coding RNA molecules that show considerable dysregulation in RCC, and they have potential for both diagnostic and prognostic applications. Research has highlighted their utility on biofluids, such as plasma, serum, and urine, in detecting RCC and characterizing its subtypes. Promising miRNA signatures have been associated with overall survival, suggesting their potential importance in the management of RCC. Exosomes, which carry a variety of molecular components, including miRNAs, are emerging as valuable biomarkers, whereas CTCs, released from primary tumors into the bloodstream, provide critical information on cancer progression. However, translation of these findings into clinical practice requires additional validation and standardization through large-scale studies and robust evidence. Although there are currently no approved diagnostic tests for RCC, the future potential of liquid biopsy in monitoring, treatment decision-making, and outcome prediction in patients with this disease is significant. This review examined and discussed recent developments in liquid biopsy for RCC, assessing both the strengths and limitations of these approaches for managing this disease.

## Simple Summary

Renal cell carcinoma (RCC) presents diagnostic challenges, especially with small renal masses, prompting research into non-invasive methods like liquid biopsy. Liquid biopsies analyze biomarkers such as microRNAs (miRNAs), exosomes, and circulating tumor cells (CTCs). miRNAs are small non-coding RNA molecules that are dysregulated in RCC and show promise in plasma, serum, and urine for diagnosis, prognosis, and subtype characterization. Specific miRNA signatures are associated with survival, highlighting their potential clinical utility. Exosomes, which carry miRNAs and other molecules, and CTCs, which provide insights into cancer progression, are also emerging as valuable biomarkers. Despite their promise, clinical application requires further validation and standardization in large-scale studies. Although no diagnostic liquid biopsy tests for RCC have been approved, these approaches hold significant potential for improving diagnosis, treatment decisions, and outcome prediction. Future research is essential to address the existing diagnostic limitations and improve RCC management.

## 1. Introduction

Renal cell carcinoma (RCC) is the most prevalent malignancy of the kidney and among the 20 most diagnosed cancer globally in 2022 [1,2]. The annual incidence of this type of cancer is estimated to be 430,000 new cases, accounting for 2.2% of all cancer diagnoses, with approximately 180,000 deaths reported each year [3]. Over the last two decades, the incidence of RCC has increased by 2% annually worldwide. The World Health Organization (WHO) categorizes RCC into various subtypes based on morphological, molecular, and genetic characteristics. Histologically, the most common RCC subtypes are clear cell RCC, which accounts for approximately 70–90% of kidney malignancies, whereas papillary RCC (types I and II) and chromophobe RCC account for approximately 10–15% and 3–5%, respectively [1]. Although hereditary renal cancer is linked to somatic mutations in the VHL gene, most cases of RCC are sporadic and lack specific genetic associations. Recent advancements in cancer genomics have revealed mutations in genes involved in epigenetic regulation, highlighting notable intra-tumor heterogeneity that may have important prognostic, predictive, and therapeutic implications [4,5].

Most patients with RCC present with no symptoms at the time of diagnosis, even in those with large tumors. Diagnosis often occurs incidentally during imaging studies, and survival outcomes largely depend on the stage at the time of detection [4,6]. Only 10% of patients present with the classic triad of symptoms, which include hematuria, flank pain, and palpable masses. Other commonly reported symptoms include fever, weight loss, and leukocytosis. Approximately 20% of patients develop various paraneoplastic syndromes, such as hypercalcemia, polycythemia, Cushing syndrome, and hypertension [1]. A contrast-enhanced, triple-phase helical CT scan is considered the gold standard for renal masses investigation, allowing the differential diagnosis between benign masses that do not require further investigation and RCC. Surgery is the preferred treatment for localized tumors, whereas chemotherapy is essential for metastatic disease. However, metastatic RCC at diagnosis is associated with a poor prognosis, with a 5-year survival rate of less than 15%. Early diagnosis is thus critical, but current diagnostic tools lack sufficient accuracy, particularly for small renal masses (SRMs) [3]. This underscores the urgent need for non-invasive screening methods and reliable biomarkers for RCC [7,8,9,10].

For this purpose, liquid biopsy is a promising approach: it is a non-invasive diagnostic tool that has gained considerable attention in recent years. Liquid biopsy is less invasive than tumor biopsy; however, to date, clinically approved liquid biopsy biomarkers for RCC diagnosis have not been reported.

**Figure 1:**
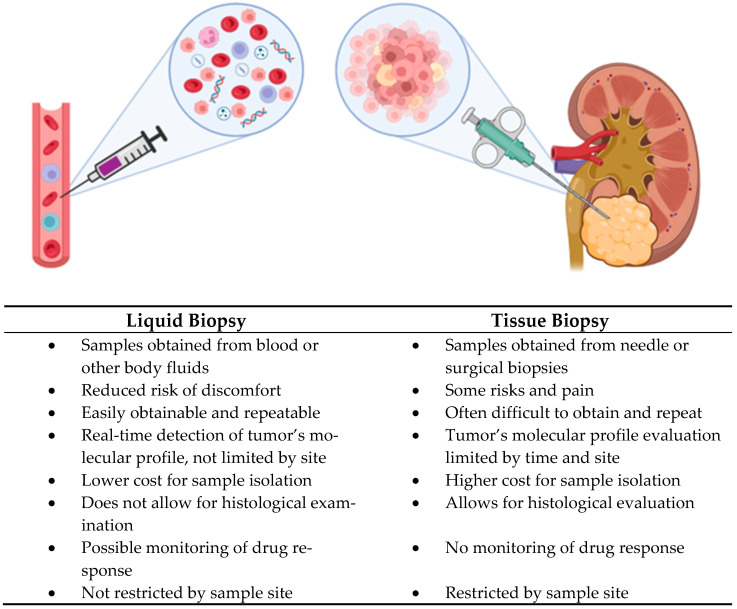
Comparison between liquid biopsy and tissue biopsy.

Comparing tissue and liquid biopsies reveals distinct approaches to cancer diagnostics, each with its specific utility in the patient care continuum [11]. Tissue biopsies, requiring physical samples from the tumor, offer a highly detailed look at the cancer’s histology and molecular genetics. This method is unparalleled in providing the definitive diagnosis and characterization of tumors, which is essential for initial treatment decisions. However, its invasiveness can be a limitation, particularly for patients with RCC due to the risk of metastatic dissemination. Liquid biopsies, conversely, offer a minimally invasive snapshot of the tumor’s current genetic landscape by analyzing circulating tumor DNA (ctDNA) or cells from a simple blood draw. This technique allows for the ongoing monitoring of tumor evolution and the detection of resistance mutations, offering a dynamic view of the disease over time. While liquid biopsies excel in accessibility and patient comfort, their sensitivity and specificity can vary based on the tumor type and stage, potentially limiting their standalone diagnostic capability (Figure 1).

The comparison between these two methodologies underscores a potential complementary relationship rather than a direct competition. Tissue biopsies provide the depth of information important for the initial diagnosis and molecular profiling, while liquid biopsies offer a broader, dynamic range of applications, particularly useful for monitoring treatment response and disease progression. The choice between them—or the decision to use both—depends on the clinical context, the specific information required, and the patient’s condition, highlighting the nuanced decision-making process in optimizing cancer care [11].

**Figure 2:**
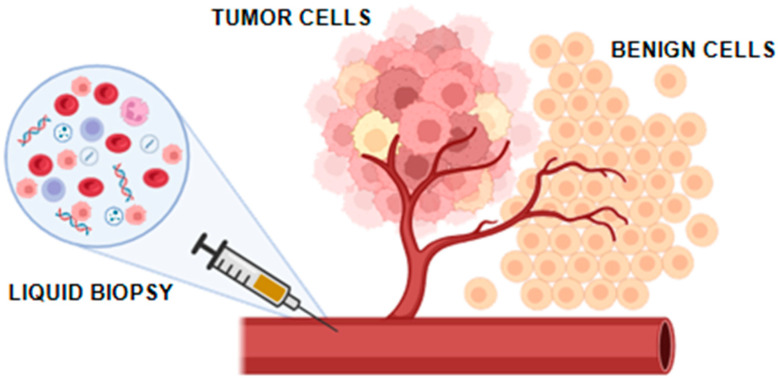
Liquid biopsy containing extracellular vesicles, circulating tumor cells and circulating tumor DNA.

Liquid biopsy involves the analysis of biofluids, such as blood or urine, to identify molecular biomarkers that provide significant information about disease characteristics. This method has already been used for the diagnosis and monitoring of various malignancies [12]. In RCC, potential biomarkers encompass circulating tumor cells (CTCs), cell-free tumor DNA (ctDNA) or circulating free DNA (cfDNA), exosomes, tumor-derived metabolites, as well as proteins found in blood and urine, as shown in Figure 2.

Here, a comprehensive review of the existing literature on liquid biopsy biomarkers for RCC has been proposed, with a particular emphasis on three promising biomarkers: microRNAs (miRNAs), exosomes, and circulating tumor cells (CTCs). The diagnostic, prognostic, and therapeutic roles of these biomarkers in RCC have been examined, addressing key clinical challenges such as early diagnosis, detection of small renal masses, and characterization of RCC subtypes. Additionally, the limitations of current technologies and methodologies in this area have been explored.

## 2. MicroRNA (miRNAs)

MicroRNAs (miRNAs) are small, non-coding RNAs that are typically composed of approximately 20 nucleotides and play a pivotal role in regulating various cellular processes, including the cell cycle, differentiation, and apoptosis. In addition, miRNAs influence multiple metabolic pathways. Notably, studies have indicated that miRNAs regulate approximately 30–60% of human genes [13]. miRNA genes are located in the introns or exons of both coding and non-coding protein genes and possess specific transcription promoters, which may be either their own or shared with other mRNAs. miRNA transcription can occur individually or in clusters, with varying expression levels across different tissues within the same organism. RNA polymerase II facilitates the transcription of miRNA genes from the genome [14,15]. The biogenesis of miRNAs occurs in two phases: a nuclear phase that generates a primary transcript and a cytoplasmic phase that involves the processing of this transcript by RNase III endonucleases DROSHA and DICER. In the cytoplasm, the primary transcript is processed into small, double-stranded miRNA/miRNA duplexes consisting of two strands designated as -3p and -5p. One strand is incorporated into the RNA-induced silencing complex (RISC), where it plays a key role in target recognition and gene silencing, while the other strand is discarded and degraded [15,16]. miRNA action is determined by its ability to bind complementary sequences in the 3′-untranslated region (UTR), thereby regulating gene expression by destabilizing mRNA or inhibiting translation. This process may involve either complete or partial pairing between miRNA and its target mRNA, leading to the degradation of mRNA or the suppression of protein translation. Through these mechanisms, miRNAs are involved in processes such as cell development, viral infections, immune responses, angiogenesis, and the progression of various cancers, including breast, lung, colorectal, ovarian, prostate cancers, and others [17,18,19,20,21,22,23,24].

Tumor cells may release miRNAs into various biological fluids, including blood plasma and serum, as well as saliva and urine. This ability makes miRNA a potential biomarker for the diagnosis and prognosis of cancer. Once secreted, miRNAs are frequently associated with protein complexes (such as Argonaute-2 or nucleophosmin) or enclosed within extracellular vesicles of varying sizes, including exosomes, microvesicles, and apoptotic bodies. These structures shield miRNAs from degradation by RNase enzymes, thereby maintaining their stability in extracellular environments. Exosomal miRNAs can influence the tumor microenvironment at distant sites by regulating gene expression and promoting tumor growth [15,25]. They not only have a close relationship with urogenital system tumors but can also reflect the miRNA expression profile of source cells. This packaging not only enhances their stability but may also reflect active tumor signaling, making them potentially more specific markers of oncogenic processes and offering distinct advantages in biomarker development.

From a diagnostic perspective, both forms of miRNA have shown potential in RCC. However, recent studies suggest that exosomal miRNAs may offer higher specificity, as they are more likely to originate from tumor cells and better reflect the molecular composition of the tumor microenvironment [7,26,27]. For instance, serum exosomal miR-210 has been associated with tumor progression and poor prognosis in clear cell RCC, demonstrating a sensitivity of 82.5% and specificity of 80.0% [28]. Furthermore, the expression of exososomal miR-146b-5p and miR-99-5p was significantly elicited to correlate with RCC patients and could be a prognostic marker for RCC [28]. These findings suggest that exosomal miRNAs could be more effective than free circulating miRNAs for certain diagnostic and prognostic purposes, although further head-to-head comparisons and validation studies are still needed.

Numerous studies have demonstrated the dysregulation of circulating miRNAs in various cancer types. Non-invasive techniques can be used to profile miRNAs in biofluids like urine, colostrum, tears, and seminal fluid, which is a significant benefit of liquid biopsy. For diagnosing and monitoring renal cell carcinoma (RCC), plasma and serum are frequently used because miRNAs remain stable in these samples. However, urine samples also present valuable opportunities for tracking miRNA levels to evaluate disease diagnosis and progression [29]. A recent study examined miRNA expression in urine for the diagnosis of clear cell renal cell carcinoma (ccRCC). The authors first analyzed miRNA expression in ccRCC specimens and healthy kidney tissues using data from public databases and then validated their findings through reverse transcription quantitative PCR (RT-qPCR) by comparing miRNA levels in ccRCC and adjacent non-cancerous tissue. They developed an algorithm to identify miRNAs likely present in the urine of patients with ccRCC, identifying miR-122, miR-1271, and miR-15b as potential markers. Validation in a cohort of 13 affected patients and 14 healthy controls revealed a sensitivity of 100% (95% CI 75–100%) and specificity of 86% (95% CI 57–98%), suggesting the potential of these urinary miRNAs as diagnostic markers, although further validation in larger studies is needed [30]. Moreover, miR-15b has also been associated with tumor resistance to targeted cancer drugs, such as sunitinib [31]. A similar study focused on papillary renal cell carcinoma (pRCC) using serum as the miRNA source. The authors aimed to determine whether miRNA dysregulation in malignant tissues could be reflected in serum samples, providing a non-invasive diagnostic method. The study also sought to differentiate between type 1 and type 2 pRCC. Serum miRNA levels were analyzed in 34 patients with pRCC type 1, 33 with pRCC type 2, and 33 control patients. This study revealed that, while differentiation between pRCC subtypes was not possible using a single miRNA marker, advanced pRCC showed elevated levels of miR-21-5p and the diagnostic accuracy improved with the inclusion of miR-210-3p. The authors proposed miR-21-5p and miR-210-3p as potential biomarkers of pRCC [32]. In another study, serum miRNAs were analyzed to identify biomarkers for RCC diagnosis. The study enrolled 296 patients (146 RCC patients and 150 healthy controls) and analyzed the serum expression of 30 miRNAs. Diagnostic efficiency was assessed using ROC curve analysis and AUC analysis, and a miRNA panel consisting of miR-224-5p, miR-34b-3p, and miR-182-5p was identified as a reliable non-invasive diagnostic biomarker for RCC, with an AUC of 0.855, demonstrating good diagnostic efficiency [33].

Recent research has also highlighted the potential of miRNA levels in biofluids for prognostic prediction in RCC. miRNA signatures have been investigated to improve prognosis prediction in patients with ccRCC using RNA-Seq data from the TCGA database. In this study, 177 differentially expressed miRNAs were identified between ccRCC and paracancerous tissues. A three-miRNA signature (miR-130b, miR-18a, and miR-223) was constructed using the LASSO Cox regression model, and patients were classified into high-risk vs. low-risk groups based on the miRNA signature. The study found a significant difference in overall survival between these groups, with a hazard ratio of 5.58 (95% CI 3.17–9.80; *p* < 0.0001), suggesting that this three-miRNA signature could be used to predict the overall survival of patients with RCC [34].

Although these findings are promising, further studies are necessary before miRNA-based tests can be approved for the diagnosis and prognosis of RCC. However, the current state of research indicates that miRNAs may play a pivotal role in the future management of RCC.

## 3. Extracellular Vesicles and Exosomes

Extracellular vesicles (EVs) can be categorized into three main subtypes based on their size, cellular origin, physicochemical properties, and biomolecular composition: apoptotic bodies, microvesicles, and exosomes. Exosomes, which are the smallest extracellular vesicles (ranging from 30 to 150 nm), are generated via the process of exocytosis. This occurs when multivesicular bodies fuse with the plasma membrane, releasing intraluminal vesicles into the extracellular space. These exosomes may carry several substances, including DNA, miRNA, mRNA, cellular metabolites, and proteins [35]. Exosome biogenesis and release are regulated by multiple factors, such as endosomal sorting complexes required for transport (ESCRT), the p53/TSAP6 pathway, Rab proteins, phospholipase D, Syndecan-syntenin-ALIX, and sphingomyelinase. The exosomal membrane is enriched with specific lipid types, including sphingomyelin, ceramide, cholesterol, and phosphatidylserine, which differentiate exosomes from other lipid-based vesicles like liposomes [36]. Exosomes serve as key mediators of intercellular communication and play a critical role in facilitating organ crosstalk. Initially overlooked and referred to as “cellular dust”, their significance as mediators of intercellular communication and disease markers, including malignancies like kidney cancer, has since been recognized. Exosomes essentially reflect the molecular profile of the producing cell, and, upon uptake by recipient cells, they can influence cell function by delivering various types of nucleic acids, proteins, or metabolites, depending on their cellular origin and biogenesis process [37]. Exosomes can be detected in several human biofluids, including blood, urine, and saliva [7].

Several techniques are available for isolating exosomes, including size-exclusion chromatography, differential ultracentrifugation, ultrafiltration, immunoaffinity capture, polyethylene glycol-based precipitation, and microfluidics [38]. Among them, differential centrifugation and immunoaffinity capture are commonly used. Differential centrifugation is favored for its simplicity and speed, although it cannot distinguish between exosomes and other impurities such as proteins or other extracellular vesicles. In contrast, immunoaffinity capture using antibodies or magnetic beads offers higher purity but is associated with lower capture rates and higher costs [27]. The unique membrane structure of exosomes protects their contents from degradation by RNases and proteases and contributes to the stability of enclosed mRNAs, miRNAs, and functional proteins. As a result, exosomes hold great potential as biomarkers for diagnosing RCC. A recent study showed that serum exosomal miR-210, which originates from tumor tissue, could serve as a novel non-invasive biomarker for clear cell RCC (ccRCC) detection and prognosis [39]. The study analyzed the expression of six miRNAs (miR-210, miR-224, miR-452, miR-155, miR-21, and miR-34a) in both tissues and serum exosomes of patients with ccRCC using RT-qPCR. Serum exosomal miR-210 was significantly upregulated in patients with ccRCC, particularly at advanced stages, with high Fuhrman grades, and in those with metastasis. This study indicated that serum exosomal miR-210 could serve as a potential biomarker for ccRCC diagnosis, prognosis, and recurrence prediction, with sensitivity and specificity of 82.5% and 80.0%, respectively.

A further in vitro study highlighted the role of exosomal miRNAs as potential biomarkers of RCC [40]. Using the 786-O ccRCC cell line and the HK-2 human renal proximal tubule cell line as controls, this study investigated exosomal miRNA content in the supernatant of both cell types. Increased levels of miR-210, miR-34a, miR-155-5p, and miR-150-5p, ranging from two- to eight-fold, were detected in exosomes from 786-O cells compared with the healthy controls. This study demonstrated that the exosomal content of miR-205 and potentially miR-150 reflected the content within the producing cells, suggesting their potential as in vivo biomarkers for ccRCC. Another study highlighted the diagnostic potential for RCC by identifying variations in miRNA expression profiles within peripheral blood exosomes when comparing RCC patients to healthy individuals [26]. Exosomal miRNA sequencing from plasma samples of five patients with RCC and five healthy controls revealed significant differences in the expression levels of hsa-mir-92a-1-5p, hsa-mir-149-3p, and hsa-mir-424-3p, which may serve as potential biomarkers for RCC diagnosis.

Moreover, exosomes have been implicated in predictions of outcome and in response to therapy in RCC, as demonstrated in a recent study [41]. The authors focused on exosomal miRNA expression profiles in venous blood samples from 35 patients with ccRCC treated with immune checkpoint inhibitors. Using RT-qPCR, they found that miRNA-146a expression increased after treatment, whereas miRNA-126 expression decreased. The combination of miRNA-146a and miRNA-126 achieved an area under the curve (AUC) of 0.752 (95% CI 0.585–0.918), with a sensitivity of 64.3% and specificity of 78.9%, suggesting the potential use of these miRNAs as biomarkers for assessing therapy effectiveness in RCC [41].

Indeed, EVs and, in particular, exosomes, have gained increasing attention as promising tools for non-invasive diagnostics in RCC. Exosomal cargo—including microRNAs, mRNAs, and proteins—has been shown to reflect tumor biology and the molecular signature of renal malignancies. Numerous studies have demonstrated that specific miRNAs encapsulated within urinary exosomes can differentiate patients with ccRCC from healthy individuals. For instance, urinary exosomal miR-126-3p, in combination with miR-449a or miR-34b-5p, has shown high diagnostic accuracy in distinguishing ccRCC cases [40]. After nephrectomy, the levels of these miRNAs return to levels comparable to those in healthy samples. Similarly, miR-542-5p was identified as a potential biomarker in von Hippel–Lindau (VHL)-associated ccRCC, with expression levels increasing in tumor-bearing patients and returning to baseline after nephrectomy [26,42]. These findings underline the importance of urinary exosome profiling, especially in hereditary cancer syndromes such as VHL disease, where early detection is crucial.

Beyond their diagnostic potential, exosomes actively contribute to RCC progression. RCC-derived exosomes promote angiogenesis by transferring pro-angiogenic molecules (e.g., VEGF, FGF2, MMP-2/9), and modulate the immune system by inducing T cell apoptosis, impairing NK cell function, and expanding regulatory T cell populations [43]. Furthermore, exosomes facilitate metastasis through epithelial–mesenchymal transition and by preparing pre-metastatic niches. They have also been implicated in drug resistance by transporting anti-apoptotic proteins and efflux pumps [44,45].

Importantly, exosomes are being explored as therapeutic carriers. Engineered exosomes loaded with tumor suppressor miRNAs (e.g., miR-34a, miR-1) [46] or immunostimulatory molecules (e.g., IL-2, PD-1 inhibitors) have shown promise in preclinical studies as targeted therapies against RCC [47,48]. Proteins such as CD105, TIMP-1, and Annexin A3 have also emerged as potential diagnostic or prognostic exosomal biomarkers [49,50,51]. Also, caveolin-1, a specific protein found in exosomes from the blood of patients with RCC, was found to be associated with a higher risk of tumor recurrence and a lower survival rate [52]. Despite these advances, further validation in large patient cohorts and the development of standardized detection protocols are required before clinical translation.

Overall, exosomes possess distinctive characteristics and the ability to carry diverse molecular components, and the evidence supports a multifaceted role of exosomes in RCC as biomarkers, drivers of pathogenesis, and vehicles for therapy. Their analysis in accessible biofluids such as urine or blood represents a minimally invasive opportunity to improve diagnosis, monitor disease progression, and develop targeted interventions.

Future research is expected to provide further insights into the clinical utility of exosomal markers in RCC.

## 4. Circulating Tumor Cells

In patients diagnosed with metastatic cancer, primary tumor cells can migrate to secondary sites via the bloodstream or lymphatic vessels. This migration is promoted by a biological phenomenon known as epithelial–mesenchymal transition (EMT). The first step in metastatic dissemination involves the invasion of the circulatory system by the cancer cells. This process, termed “cellular seeding”, allows cancer cells to spread to distant parts of the body, where they can establish secondary tumor sites [53]. During metastasis, circulating tumor cells (CTCs) are released from the primary tumor into the bloodstream. Detecting and analyzing these CTCs in blood samples have significant potential for cancer diagnosis. As CTCs are derived directly from the primary tumor, their presence in the bloodstream is theoretically specific to the tumor of origin. Thus, the identification and characterization of CTCs in blood samples offer critical insights into cancer diagnosis, monitoring of disease progression, and evaluation of treatment efficacy [54]. However, despite these advantages, CTC detection is not common, particularly in patients with small tumors. The absence of CTCs in early-stage tumors limits their diagnostic utility. Furthermore, the detection of CTCs in blood is challenging because of their morphological similarities with white blood cells, thereby making differentiation difficult. Nonetheless, both CTCs and white blood cells are important for monitoring the prognosis of renal cell carcinoma (RCC).

A recent study examined the prognostic value of total CTCs and circulating tumor-cell-associated white blood cell (CTC-WBC) clusters, in addition to cancer size in patients with RCC [55]. The study, which included 163 RCC cases, found that higher counts of CTCs and CTC-WBC clusters, along with larger solid tumor diameters, were negative prognostic factors correlated with poorer metastasis-free survival. This study demonstrated a negative correlation between CTC count, solid tumor size, and overall survival in patients with RCC [55]. Various methods are employed to enrich the CTC population, including mechanical-based approaches (e.g., cellular size and density) and antibody- or fluorescence in situ hybridization (FISH)-based techniques. Mechanical methods, such as cell size-based sorting, are widely used, but the overlap in size characteristics between CTCs and WBCs complicates differentiation [56,57]. To address this issue, more refined techniques have been developed, including antibody-based enrichment strategies that rely on specific antigens present on the cell surface, allowing for the positive or negative selection of cell populations. A negative selection method uses the leukocyte marker CD45 and its specific antibody to remove leukocytes from samples, resulting in the enrichment of the circulating tumor cell (CTC) population [58]. Alternatively, positive enrichment employs immunomagnetic beads or nanochips coated with antibodies that target common circulating tumor cell (CTC) markers. The epithelial cell adhesion molecule (EPCAM) is a notable marker and is the only FDA-approved marker for diagnosing breast, colon, and prostate cancers [59,60]. Once isolated, CTCs can be used to diagnose and monitor various cancers, including colorectal, bladder, lung, and prostate cancers [61,62,63,64].

CTCs have been investigated in RCC for over two decades, yet their clinical implementation remains limited. One of the main challenges is the low expression of epithelial markers such as EpCAM in RCC, which reduces the sensitivity of traditional detection systems like CellSearch [65,66]. Alternative platforms, including microfluidic devices (e.g., RUBYchip), label-free systems, and immunomagnetic techniques using CA9 and CD147 antigens, have shown improved sensitivity for CTC isolation and characterization in RCC patients [67,68,69].

Several studies have explored the prognostic value of CTCs in localized and metastatic RCC. In a Chinese cohort, the NanoVelcro system effectively captured CTCs and identified associations with mesenchymal markers (e.g., Vimentin) and clinical stage [69]. An Italian study using CellSearch^®^ (Menarini, Bologna - Italy) on 195 mRCC patients showed that individuals with ≥3 CTCs had significantly shorter overall survival (OS) and progression-free survival (PFS) compared to those with <3 CTCs [67,68]. Another US-based study using quantitative microscopy found that patients with high CTC trajectories had reduced OS [70].

The preoperative and postoperative dynamics of CTC counts have also been evaluated. In a study using the SDI-Chip, CTC numbers dropped significantly after nephrectomy in early-stage RCC, and preoperative CTC counts were closely associated with tumor stage and malignancy, suggesting a potential role in prognostication.

Although data on CTCs for renal cancer diagnosis are limited, a recent study suggested that baseline CTC count could serve as a prognostic indicator of metastatic RCC. The study concluded that the presence of three or more CTCs per milliliter at baseline was linked with significantly shorter progression-free survival and overall survival in patients with metastatic RCC, who were receiving first-line antiangiogenic tyrosine kinase inhibitors, particularly sunitinib, sorafenib, and pazopanib. However, it is important to emphasize that factors such as patient age and the type of first-line therapy chosen can also impact survival outcomes in malignancies [67,68]. The prognostic role of CTCs was further explored in a study which investigated the dynamic changes in CTCs and Beclin-1 expression in RCC prognosis [71]. Beclin-1 is an autophagy-related gene, and its expression is variable in CTCs. The study enrolled 69 patients with RCC and divided them into metastasis-free and metastatic groups based on postoperative status. Multiple CTC tests were performed, and peripheral blood samples were collected at three time points: preoperative, 6 months, and 12 months postoperatively. CTCs were categorized into epithelial, mesenchymal, and mixed phenotypes based on surface biomarkers. Although initial CTC counts did not differ significantly between the metastasis-free and metastatic groups, the metastatic group showed a significant increase in mixed CTCs at 12 months compared to earlier time points. Furthermore, Beclin-1-positive CTCs were significantly more prevalent than Beclin-1-negative CTCs preoperatively in the metastatic group (*p* < 0.05). The study concluded that RCC recurrence or metastasis was associated with dynamic changes in the CTCs, particularly mesenchymal CTCs and Beclin-1-positive CTCs.

Despite this progress, results across studies are still heterogeneous. A systematic review highlighted the lack of standardization and the limited reproducibility of CTC detection methods in RCC [72]. Moreover, while several studies support an association between CTCs and adverse features [69,73,74] others failed to show significant prognostic relevance [75,76]. The epithelial–mesenchymal plasticity of RCC cells complicates the detection and clinical interpretation of CTCs.

Nevertheless, recent advances indicate that CTC enumeration and molecular profiling may provide dynamic biomarkers for disease monitoring and treatment response, particularly when integrated with other liquid biopsy components such as cfDNA or exosomal RNA [71,77,78].

Currently, no specific diagnostic assays have been approved for RCC, primarily due to the lack of well-established and specific markers for RCC and its subtypes. As a result, CTCs could play a crucial role in the future, particularly in follow-up care and therapeutic decision-making. Future studies should focus on standardizing detection protocols, validating results in larger cohorts, and exploring multi-marker liquid biopsy strategies, exploiting the fact that CTCs could predict outcomes and prognosis in patients with renal cancer.

## 5. Circulating Tumor DNA

Circulating tumor DNA (ctDNA) has emerged as a promising biomarker for both the diagnosis and monitoring of renal cell carcinoma (RCC), positioning itself as a key component of liquid biopsy. This non-invasive approach complements traditional imaging techniques and offers significant potential for detecting genetic alterations, including mutations in the von Hippel–Lindau (VHL) gene or changes in genes related with the mTOR pathway [75,79,80]. ctDNA, which encompasses fragmented DNA shed by tumor cells into the bloodstream, is closely related to the genetic profile of the tumor and can offer significant insights into its molecular features.

The effectiveness and reliability of ctDNA analysis depend on the methods used for its isolation. These can be broadly categorized into non-commercial methods, which utilize time-consuming yet highly efficient extraction techniques, and commercial methods, which prioritize repeatability and reproducibility but typically yield lower quantities of ctDNA [81,82]. In the early stages of ctDNA research in RCC, several studies have compared the presence of ctDNA between patients with RCC and healthy controls, using tumor tissue DNA as a reference. In a recent study, the authors detected ctDNA in only two out of five patients with RCC [83], while another study identified ctDNA in one of nine patients with RCC [84]. Despite the initially low detection rates, subsequent studies, particularly those involving metastatic and non-metastatic patients and incorporating deep sequencing techniques, reported detection rates ranging from 42% to 54% [85,86,87]. Targeted sequencing of plasma DNA eliminates the need for prior tumor tissue analysis and allows for the identification of newly acquired mutations, although this method exhibited lower sensitivity compared with tumor-guided approaches. Most of these studies, particularly those on metastatic RCC, reported ctDNA detection rates exceeding 50%, with a strong correlation between tumor burden and ctDNA detection [88,89,90]. This correlation was confirmed in a further study, which reported a relatively low detection rate of 33% in patients whose plasma samples were collected after primary tumor removal [91].

Beyond its diagnostic potential, ctDNA can be used to monitor disease progression and treatment response in RCC. By tracking changes in the quantity and genetic profile of ctDNA over time, clinicians can assess tumor dynamics, which may drive therapeutic interventions. Furthermore, ctDNA analysis facilitates early detection of disease recurrence or metastasis, allowing for timely modifications to treatment regimens and potentially improving patient outcomes. In a study, ctDNA levels were reported to increase prior to the initiation of treatment and decreased with treatment response, only to rise again with disease progression or lack of treatment efficacy [85]. Similar findings were reported in further studies who conducted longitudinal ctDNA assessments in patients with RCC [87,92]. Notably, these studies indicated that ctDNA detection in patients at various stages of RCC was significantly linked with an increased risk of death and shorter progression-free, cancer-specific, and overall survival [92,93,94].

In a recent study, ctDNA was used to assess the response of metastatic RCC patients to immune checkpoint inhibitors. The study found that ctDNA levels decreased in patients who responded to treatment or showed partial responses, whereas ctDNA levels increased in those with disease progression [95]. More recently, ctDNA has transitioned from a diagnostic and monitoring tool to a potential predictor of disease outcomes. For instance, a further study involving 48 patients who underwent partial nephrectomy for T1a RCC, ctDNA was detected in over 75% of patients who experienced upstaging to T3a disease, compared with only 2.8% in patients with confirmed T1a disease [96].

The use of ctDNA as a non-invasive biomarker of RCC is rapidly advancing. With its rapid, cost-effective, and minimally invasive nature, ctDNA is expected to play an increasingly important role at various stages of RCC, from early diagnosis to progression prediction and treatment monitoring. Ongoing studies are focused on evaluating the potential of ctDNA in RCC, with the goal of translating research findings into routine clinical practice [97]. However, challenges remain in optimizing technical assays, maintaining high-quality results, and improving library preparation methods to achieve sufficient sequencing depth, particularly for methylation profiling.

In addition to quantifying ctDNA levels and analyzing their fragment length, the detection of specific genetic and epigenetic alterations is gaining increasing relevance in the context of RCC. While *VHL* inactivation is a well-established initiating event ccRCC, it is insufficient alone to cause tumorigenesis, as evidenced by its low penetrance in germline mutation carriers and lack of tumor formation in Vhl-deficient mice [81,82,98]. Additional mutations—particularly in genes involved in chromatin regulation such as *PBRM1*, *SETD2*, *BAP1*—are now recognized as critical contributors to ccRCC development and progression [99,100,101]. *PBRM1* truncating mutations are found in up to 41% of cases, making it the second most commonly mutated gene after *VHL* [102], while *BAP1* and *SETD2* mutations are associated with aggressive histological features, higher tumor grades, and poorer survival outcomes [103,104]. Notably, some mutations—such as those in *BAP1* and *PBRM1*—occur mutually exclusively, suggesting distinct molecular subtypes of ccRCC with prognostic relevance [105].

These genes are all located on chromosome 3p, near the *VHL* locus, and are often co-deleted in the characteristic 3p loss found in over 90% of ccRCC cases. This spatial and functional proximity underscores the importance of chromatin dysregulation as a unifying theme in ccRCC pathogenesis [105]. Emerging studies using ctDNA have begun to detect these mutations non-invasively: targeted sequencing panels covering hundreds of cancer-related genes have successfully identified *VHL*, *PBRM1*, and *BAP1* alterations in plasma samples from patients with metastatic RCC, with concordance rates up to 77% compared to matched tissue samples [81,82]. These findings highlight the potential of ctDNA not only for tumor monitoring but also for molecular subtyping. Additionally, structural variants such as chromosomal translocations, more often observed in non-clear cell RCC, could potentially be captured by comprehensive ctDNA profiling. In parallel, ctDNA assays integrating epigenetic signatures—such as methylation profiling—are under development and may further enhance the sensitivity of liquid biopsy in early-stage RCC.

Circulating and exosomal miRNAs have shown high stability in body fluids and consistent correlation with tumor biology, making them promising candidates for early detection and prognosis [7,15,16]. ctDNA, although technically more challenging in early-stage RCC due to low release, offers high specificity and may be particularly useful in advanced disease settings for monitoring and mutation tracking [15,86,87] and for providing dynamic, real-time insights into tumor biology. In contrast, despite long-standing research, the clinical use of CTCs in RCC remains limited due to technical difficulties in their isolation and the absence of RCC-specific surface markers [3]. Therefore, miRNAs and ctDNA currently appear to be the most promising tools in the management of RCC.

## 6. Other Potential Protein, Metabolic, and Molecular Biomarkers for Diagnosis and Monitoring of Renal Cell Carcinoma

As mentioned, one of the major goals in cancer research is the identification of molecular markers in body fluids like blood and urine. These markers can help with screening, diagnosing, monitoring treatment, and following up with RCC patients. In addition to the biomarkers already discussed, many new molecular, protein, and metabolic markers are now emerging. Oxidative stress, inflammation and hypoxia can promote the development and progression of cancers. These stress conditions are associated with the release of stress proteins. Among them, high levels of heat shock protein 27 (Hsp27) have been found in the blood and urine of patients with high-grade RCC, as reported by White et al. [106]. Moreover, pro inflammatory cytokines, including IL-1, IL-6, and TNF-α, are able to regulate the production of other inflammatory mediators which play a role in the recruitment of immune cells to sites of inflammation. Studies showed that blood levels of an inflammatory mediator, serum amyloid A (SAA) have a positive correlation with tumor stage, especially in more advanced cases [107,108]. Recently, it has been shown that both innate and adaptive immune cells are recruited into RCC tumors and become activated by tumor-associated antigens. However, the tumor often manages to evade immune attacks through mechanisms that are not yet fully understood. One of the most well-characterized of these is the T-cell inhibitory pathway regulated by the protein programmed death 1 (PD-1). Normally, PD-1 plays a key role in controlling immune responses and preventing excessive inflammation and tissue damage. It is believed that, as an adaptive mechanism, tumor cells may express PD-1’s ligand (PD-L1/B7-H1) to suppress the host’s immune response and promote immune escape. Notably, the administration of an anti-PD-L1 was demonstrated to slow the progression in patients with advanced tumors [109].

Another key player in the development of cancer is the disruption of the normal process of apoptosis. Toyama et al. found that RCC patients had significantly lower levels of tumor necrosis factor-related apoptosis-inducing ligand (TRAIL) in their blood before surgery compared to healthy controls, and this reduction was linked to patient survival [110]. Furthermore, neutrophil gelatinase-associated lipocalin (NGAL), kidney injury molecule-1 (KIM-1), matrix metalloproteinases (MMPs), and aquaporins urinary levels have been found to be significantly influenced by cancerous kidney diseases [111]. 

Many peptides play roles in normal physiological functions, and some of them may serve as valuable biomarkers in the context of renal cell carcinoma (RCC). Proteomic research has increasingly turned to mass spectrometry as a powerful method for analyzing these peptides and proteins. Notably, Authors reported an upregulation of peptides related to beta-tubulins, zinc finger proteins, and RNA-binding proteins in RCC patients. The authors suggested that these three peptide peaks could potentially be used not only for the detection of RCC, but also as markers for monitoring treatment response [112]. 

Metabolomic analysis has been used as another a powerful toll to study metabolites in biological samples, such as tissues, urine or blood, leading to the identification of many metabolites that may be considered potential RCC biomarkers, including hydroxybutyrylcarnitine, decanoylcarnitine, carnitine, dodecanoylcarnitine, and norepinephrine sulfate, which were found in much higher concentrations in cancer tissues and in the urine of cancer patients, supporting the fundamental role that metabolism plays in the development and progression of cancer [113,114,115]. Moreover, studies demonstrated that nuclear magnetic resonance-based metabolomic can be considered a powerful tool for differentiating between various subtypes of kidney cancer. By examining the metabolic profiles of tumor samples, it is possible to identify distinct patterns in metabolite concentrations that correspond to specific renal cancer types, such as clear cell RCC, papillary RCC, and chromophobe RCC [116,117]. Furthermore, a growing body of evidence has shed lights on the roles of a small group of seemingly harmless metabolites that, when abnormally accumulated, are converted into oncometabolites that have a range of properties that can promote tumorigenesis and tumor progression. Several oncometabolites involved in renal cancer strongly impact chromatin remodeling and epigenetic alteration, including L-2-hydroxyglutarate, succinate, and fumarate. These changes contribute to an epithelial–mesenchymal transition and a pseudohypoxic signature, both of which are associated with the aggressive characteristics of various RCC subtypes [118].

Given the potential of these biomarkers in reflecting real-time changes in tumor behavior, they could become invaluable in monitoring treatment responses, predicting disease outcomes, and ultimately improving patient care in RCC (Table 1).

## 7. Microbiota in Renal Cell Carcinoma

Recent studies have underlined the role of gut microbiota (GM) as a contributor to the pathogenesis of RCC, with specific bacterial populations which are linked to both increased and decreased risk of disease. The GM plays a significant role in modulating inflammation, DNA damage pathways, and the production of oncogenic metabolites, all of which are implicated in cancer development. Alterations in the gut microbiome can lead to the release of microbial DNA (cmDNA) into the blood, resulting in detectable levels of cmDNA [119]. While most studies have focused on cancers such as lung, colorectal, and liver cancer, the principles of cmDNA detection via liquid biopsy are applicable to RCC. Given the role of the GM in modulating systemic inflammation and immune responses, alterations in the microbiome may influence RCC pathogenesis and progression [121]. The GM can influence RCC development through the modulation of the levels of important metabolites, including short-chain fatty acids and dietary amino acids, such as glutamine, tryptophan, and arginine. Studies observed that the GM contributes to RCC metastasis through kynurenine, a tryptophan-derived metabolite, which influences many cellular processes such as proliferation, migration, apoptosis, adhesion, and differentiation. Moreover, it has been shown that trimethylamine oxide (TMAO), a renal toxin produced from microbial metabolites, accelerates renal tubular-interstitial fibrosis and exacerbates kidney dysfunction. These findings have broadened our understanding and opened up new possibilities for the diagnostic and therapeutic applications of microbiota [120]. Chen et al. analyzed GM from ccRCC patients identifying notable differences in the GM composition compared to healthy individuals. Specifically, they found five bacterial genera Blautia, Streptococcus, [Ruminococcus]_torques_group, Romboutsia, and [Eubacterium]_hallii_group positively associated with ccRCC. Furthermore, Streptococcus lutetiensis was found to promote proliferation and migration of ccRCC via the TGF-signaling pathway [120]. Moreover, the genitourinary tract was long believed to be sterile; however, emerging studies support the presence of a specific urinary microbiome linked to many pathological conditions, including kidney cancer [122]. Furthermore, microorganisms associated with tumors, also known as tumor microbiota, are now recognized to have a key role in the tumor microenvironment. Based on these observations, Authors identified three predominant bacterial DNA signatures: Bacteroidia, TM7-1, and Sphingomonadales, within extracellular vesicles collected from patients with RCC. Based on these findings, the authors created a BTS index which demonstrated promising potential as a reliable oncometscreening tool for RCC [123]. Kovaleva et al. observed a significant correlation between tumor microbiota and the content of PU.1+ macrophages and CD66b+ neutrophils in RCC. Specifically, the number of PU.1+ cells and CD66b+ cells along with a high bacterial tumor load was significantly associated with poor prognosis [124]. In addition, immune cell responsiveness has been associated with GM, as demonstrated by Ninkov et al., that observed an improved function of peripheral blood mucosa-associated invariant T cells in metastatic RCC after receiving fecal microbiota transplantation, enhancing anti-tumor immunity [125]. 

Conclusively, the regulation of cell metabolic and immunological functions by gut and tumor microbiota can exert several effects on RCC; understanding the interactions between them could pave the way for developing a personalized screening system and enhancing the prevention and treatment of RCC.

## 8. Conclusions and Future Perspectives

Despite recent advancements, the diagnosis of renal cell carcinoma (RCC) remains challenging, particularly in patients with small renal masses. Improved RCC diagnosis is essential for better patient outcomes and increased chances of successful treatment. This underscores the need for non-invasive screening tools and biomarkers, prompting the exploration of liquid biopsy approaches, holding considerable promise for achieving earlier detection and real-time monitoring of renal tumors. The use of circulating biomarkers such as microRNAs (miRNAs), exosomes, ctDNA, and circulating tumor cells (CTCs) can provide valuable insights into tumor dynamics, helping guide both diagnosis and treatment decisions. miRNAs, small non-coding RNAs, are frequently dysregulated in RCC, thus offering a significant diagnostic and prognostic potential. Numerous studies have highlighted the utility of miRNAs in various biofluids, such as plasma, serum, and urine, for the detection of RCC and differentiation of its subtypes. Promising miRNA signatures have been identified, which are correlated with patient outcomes and overall survival, thus emphasizing their potential role in the management of RCC.

Similarly, exosomes, which carry a diverse array of molecules, including miRNAs, have emerged as promising and particularly intriguing biomarkers for RCC, not only as a source of diagnostic and prognostic markers, but also as potential vehicles for drug delivery, due to their natural ability to target specific cells and protect molecular cargo. The integration of liquid biopsy into RCC clinical management is still in its early stages, but several components are progressing rapidly. Circulating and exosomal miRNAs emerge as technically feasible and reproducible, with good detection in multiple biofluids. ctDNA, although currently limited by low shedding in early-stage RCC, shows promise for advanced disease profiling and therapeutic monitoring. Additionally, CTCs, which shed from primary tumors into the bloodstream, offer significant insights into tumor biology and metastatic potential. Even though detecting CTCs remains challenging, recent advances, such as label-free enrichment and single-cell technologies, are bringing new opportunities for their study.

Although these liquid biopsy approaches are promising, their clinical application requires further large-scale prospective validation studies and standardization of detection protocols. Key challenges include the need for larger, more robust studies and the identification of specific biomarkers for reliable diagnosis and monitoring. Currently, no diagnostic assays have been approved specifically for RCC; however, liquid biopsy has the potential to play a significant role in follow-up care, therapeutic choices, and outcome prediction in patients with RCC. In parallel, engineered exosomes may serve both diagnostic and therapeutic functions, opening new avenues for personalized and targeted therapy. As liquid biopsy techniques continue to evolve and integrate into precision oncology, they offer a compelling opportunity to revolutionize RCC care, leading to earlier detection, more accurate risk stratification, and ultimately improved patient outcomes and quality of life.

## Figures and Tables

**Table 1:** Potential biomarkers from biofluids.

Biomarker	Type	Sample	Clinical Relevance	Reference(s)
miR-122	Molecular	Urine	Promotes malignant phenotypes	Cochetti, G. et al. [30]
miR-1271	Molecular	Urine	Altered expression in RCC patients	Cochetti, G. et al. [30]
miR-15b	Molecular	Urine, Tissue	Anti cancer therapy resistance	Cochetti, G. et al. [30] Lu et al. [31]
miR-21-5p/miR-210-3p	Molecular	Serum	Potential biomarkers of pRCC	Kalogirou, C. et al. [32]
miR-224-5p/miR-34b-3p and miR-182-5p	Molecular	Serum	Potential biomarkers of pRCC	Huang, G. et al. [33]
miR-130b/miR-18a/miR-223	Molecular	Serum	Prediction of the overall survival of patients with RCC	Luo, Y. et al. [34]
Exosomes	Molecular, protein	Serum, Tissue	Cancer detection, prognosis and response to therapy	Wang, X. et al. [39] Crentsil, V.C. et al. [40] Xiao, C.-T. et al. [26] Ivanova, E. et al. [41]
Circulating tumor cells	Whole tumor cells	Serum	Negative prognostic factor, diagnosis and monitoring	Guan, Y. [55]
Circulating tumor DNA	Tumor-derived DNA fragments	Plasma	Biomarker for both the diagnosis, monitoring and response to therapy	Hahn, A.W. et al. [88] Pal, S.K. [89] Maia, M.C. [90] Park, J.S. [96]
Hsp27	Protein	Serum, Urine	Potential biomarkers of RCC	White, N.M.A. et al. [106]
SAA	Protein	Serum	Positive correlation with tumor stage	Steffens, S. et al. [107] de Martino, M. et al. [108]
PD-L1	Immune checkpoint protein	Tumor tissue, Blood	Immune escape; target for immunotherapy	Ngo, T.C. et al. [109]
TRAIL (TNF-related apoptosis-inducing ligand)	Apoptotic mediator	Blood	Decreased in RCC; correlates with survival	Toiyama, D. et al. [110]
NGAL, KIM-1, MMPs, Aquaporins	Kidney injury and matrix markers	Urine	Altered levels in RCC	Pastore, A.L. et al. [111]
β-Tubulin peptides, Zinc Finger, RNA-binding proteins	Peptides Proteins	Serum	Upregulated in RCC; potential for diagnosis and monitoring	Yang, J. et al. [112]
Hydroxybutyrylcarnitine, Decanoylcarnitine, Carnitine, Dodecanoylcarnitine, Norepinephrine sulfate	Metabolites	Urine, Tissue, Blood	Elevated in RCC; identified via metabolomics	Nizioł, J. et al. [113] Monteiro, M.S. et al. [114] Arendowski, A. et al. [115]
Oncometabolites (L-2-hydroxyglutarate, Succinate, Fumarate)	Metabolites	Tumor tissue, Blood	Promote tumor progression; associated with aggressive subtypes	Yong, C. et al. [118]
NMR-based Metabolomic Profile	Metabolomic Signature	Tissue, Urine, Blood	Altered expression in RCC patients	Nizioł, J. et al. [116] Zira, A.N. et al. [117]
Microbial DNA	Microbial-derived DNA fragments	Plasma	RCC development and metastasis	Leonard, S. et al. [119] Wu, K. et al. [120]

## Data Availability

No new data were created or analyzed in this study. Data sharing is not applicable to this article.

## Abbreviations

The following abbreviations are used in this manuscript:

ccRCC	clear cell Renal Cell Carcinoma
cfDNA	circulating free DNA
CTCs	Circulating Tumor Cells
ctDNA	cell-free tumor DNA
EV	Extracellular Vesicles
FISH	Fluorescence In Situ Hybridization
miRNAs	microRNAs
RCC	Renal Cell Carcinoma
SRMs	Small Renal Masses
VHL	Von Hippel Lindau
WBC	White Blood Cells
WHO	World Health Organization
